# Assessment of apoptosis and appearance of hepatocyte growth factor in placenta at different gestational ages: A cross-sectional study

**DOI:** 10.18502/ijrm.v19i6.9372

**Published:** 2021-07-27

**Authors:** Ilze Kreicberga, Anna Junga, Māra Pilmane

**Affiliations:** Institute of Anatomy and Anthropology, Rīga Stradiņš University, Riga, Latvia.

**Keywords:** Pregnancy, Placenta, Gestational age, Apoptosis, Immunohistochemistry.

## Abstract

**Background:**

Fetal growth is determined by the interaction between mother and fetus using the placental interface throughout the pregnancy.

**Objective:**

To research apoptosis and appearance of hepatocyte growth factor (HGF) in placentas of different gestational ages and to describe the anthropometrical and clinical indices of mothers and newborns.

**Materials and Methods:**

The study material was obtained from 53 human immunodeficiency virus negative pregnant women of legal age without systemic diseases. The staining of placental apoptotic cells was processed by a standard in situ cell death detection kit. The detection of HGF was provided by the ImmunoCruz goat ABC Staining System protocol sc-2023. Relative distribution of positive structures was evaluated using the semiquantitative counting method.

**Results:**

The mean rank value of the amount of HGF-containing cells (cytotrophoblasts, syncytiotrophoblasts, extravillous trophoblasts, Höfbauer cells, and cells of extraembryonic mesoderm) was 1.61 ± 0.94. Apoptotic cells (cytotrophoblasts, syncytiotrophoblasts, extravillous trophoblasts, and cells of extraembryonic mesoderm) were found in all placental samples of various gestational ages (term 13.00 ± 13.05 and preterm 27.00 ± 18.25); in general, their amount decreased with advancing gestational age of the placenta (p < 0.01).

**Conclusion:**

Weight of a placenta directly depends on the gestational age and correlates with the main fetal anthropometrical parameters (weight, length, and head and chest circumferences). The decrease in HGF-containing and apoptotic cells with advancing gestation depends on the adaptation potential of the placenta, proving the other ways of cellular disposition.

## 1. Introduction

Fetal growth and development is determined by the interaction between mother and fetus using the placental interface throughout the pregnancy. Placental insufficiency is associated with increased perinatal mortality and morbidity (1). Understanding the physiological and pathological processes in the placenta may improve the problem-solving ability and the outcome for the fetus.

Apoptosis, programmed cell death, is a normal part of tissue turnover and an essential feature of placental development (2). During pregnancy, the proliferation and apoptosis of trophoblasts are closely regulated in a dynamic balance (3). At the beginning of pregnancy, apoptosis affects maternal immune cells, enhancing the tolerance of fetal allografts (4). Further, into the pregnancy, it plays a significant role in the morphogenesis and the development of the placenta (5, 6), contributing to the growth and maturation of the villi. The amount of apoptosis in placental villi is lowest in the first trimester and increases with placental growth and advancing gestation (2). Even the relative number of apoptotic cells compared to the total cell number in the placental villi correlates with the day of gestation (7). Apoptosis is a normal part of placental development, but also is suggested to be a significant regulator in placental dysfunction. Apoptosis may be included in the pathophysiology of diseases (8) such as miscarriage, pre-eclampsia, and intrauterine growth restriction (IUGR) (9, 10). Higher appearance of macrophage infiltration and trophoblast apoptosis have been detected in the placental villous tissues in women with recurrent miscarriages (11).

Migration of trophoblast is regulated by several cytokines and growth factors. Hepatocyte growth factor (HGF) plays a significant role in placental development and growth. It is secrated by extravillous trophoblast, syncytiotrophoblast, endothelial and mesenchymal cells (12). Complicated pregnancies are associated with low levels of HGF in placentas (13).

The aim of this study was to research programmed cell death and the appearance of HGF in preterm and term placentas of different gestational ages and to describe the anthropometrical and clinical indices of mothers and newborns for the identification of the most important diagnostic and prognostic factors of placental status and fetal well-being.

## 2. Materials and Methods

In this cross-sectional study, 53 human immunodeficiency virus-negative pregnant women of legal age (> 18 yr) without systemic diseases, having sufficient antenatal care were included. Women, who refused from the participation in the study, were excluded.

Maternal and placental data were obtained from the medical records of the Riga Maternity Hospital, Riga, Latvia. The data included women's maternal age, pre-pregnancy body weight, height, calculated body mass index (BMI), number of previous pregnancies and childbirths, gestational weeks at delivery, and placental weight.

Anthropometrical parameters of neonates were obtained from the medical records. Birth weight, body length, head and chest circumferences, calculated ponderal index (PI = 100 × body mass (gr)/height (cm)${}^{3}$) were noted.

Patients were divided into two groups:

Group (I): Term study group including 19 cases of term deliveries.

Group (II): Preterm group including 34 preterm delivery cases from 22 to 36 wk of pregnancy with various outcomes.

### Sample collection

Sampling for morphological studies was collected at the Riga Maternity Hospital, Riga, Latvia from May 2009 to September 2011.

**Figure 1:**
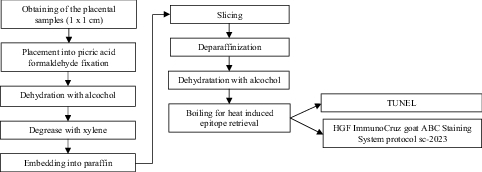
Processing of the placental samples. TUNEL: Terminal deoxynucleotidyl transferase dUTP nick end labeling, HGF: Hepatocyte growth factor.

Two 1 × 1 cm samples were taken from symmetrically located areas of the chosen placentas through all the layers of the placental tissues using a single-use surgical knife and placed into a picric acid-formaldehyde fixation, a technique originally described more than 50 yr ago (14). Samples were labeled with the assigned study number and were taken to the Institute of Anatomy and Anthropology of the Riga Stradins University, Riga, Latvia for further processing (Figure 1).

### Immunohistochemistry

Staining of apoptotic cells by terminal deoxynucleotidyl transferase dUTP nick end labeling (TUNEL) was processed by a standard in situ cell death detection kit, POD Cat. No 11684817910, manufactured by Roche Diagnostics, in a working dilution of 1:10 (15). Processing of samples for application of polyclonal HGF goat antibodies, manufactured by R&D in a working dilution of 1:300, was provided by the ImmunoCruz goat ABC Staining System protocol sc-2023 (Santa Cruz Biotechnology, Inc., 2011).

Negative and positive controls were provided to avoid background staining and non-specific binding of secondary antibodies. Primary antibodies were omitted for the negative control, obtaining preparations with no staining. Tissues known to contain molecular factors, either from the manufacturer's recommendations or from previous studies conducted at the Institute of Anatomy and Anthropology, were used as positive controls.

The immunohistochemistry findings were evaluated semiquantitatively by the amount of positive indicator cells or extracellular matrix (ECM) structures in a visual field (16): none 0, occasional 0/+ [0.5], few + [1], moderate ++ [2], numerous +++ [3], and abundant ++++ [4]. The evaluation was performed after complete observation of both samples of each placenta. The number of positive structures was detected in 10 visual fields, and the rounded average was stated as the finding of the sample.

### Ethical considerations

The research work was done in accordance with the Declaration of Helsinki. The study was approved by the Ethics Committee of the Riga Stradins University, Riga, Latvia. All participants signed an informed consent form prior to the study.

### Statistical analysis

To characterize the research groups, descriptive statistic methods were used. For the description of each marker, mean and standard deviation were used. For the comparison of groups, Mann-Whitney U-test was used (17). To evaluate the cross-compliance of two variables, Pearson's correlation coefficient (r) was calculated (18). Pearson's correlation was chosen due to the normal distribution of the data analyzed. The reported statistical significance was set at p < 0.05, while p < 0.01 was reported as statistical significance sometimes for more comprehensive research purposes. Statistical analysis was conducted using the Statistical Package for the Social Sciences (SPSS) program, version 23.0 (IBM Corporations, USA).

## 3. Results

The study included 53 women of various age, number of pregnancies and childbirths (Table I), and gestational age at the time of delivery (varying from 22 to 40 wk). The pre-pregnancy maternal BMI varied from 17.2 to 36.6 kg/m${}^{2}$ with a mean BMI of 23.74 ± 4.72 kg/m${}^{2}$.

In total 34 neonates were premature, born between 22 and 35 wk of gestation, with weights ranging from 540 to 2390 gr; 19 term neonates were born between 37 and 40 wk of gestation, with weights ranging from 2740 to 4630 gr. The median time of gestation was 33.06 wk ± 5.09. Table II presents the mean anthropometrical parameters of the study neonates.

Neonatal anthropometric parameters of the entire sample, including body weight, body length, and head and chest circumference, were directly proportional to the gestational age.

Fifty-three placentas had no remarkable anomalies, with weights ranging from 220 to 930 gr, a mean weight of 448.95 ± 157.50 gr. The differences between the values of the mean weight of preterm (401.81 ± 133.54 gr) and term placentas (637.50 ± 91.924 gr) were statistically significant (p < 0.01). When comparing the placental weight with maternal parameters such as age, pregnancy, number of childbirths, weight, length, and BMI before the actual pregnancy and weeks of gestation in the entire study, we found statistically significant positive correlations of the placental weight with maternal weight before (r = 0.342, p = 0.04) and weight gain during the pregnancy (r = 0.342, p = 0.04). In addition a strong correlation was found between the placental weight and weeks of gestation (r = 0.537, p < 0.01).

The amount of HGF-containing cells (cytotrophoblast, syncytiotrophoblast, extravillous trophoblast, Höfbauer cells, and cells of extraembryonic mesoderm) in the placentas did not correlate with the gestational age of placenta and appeared from none (0) to abundant (++++) per visual field. The mean rank value of the amount of HGF-positive cells was 1.61 ± 0.94. We found a statistically significant negative correlation between the rank value of HGF and the maternal weight gain during pregnancy (r = -0.301; p = 0.032).

**Figure 2:**
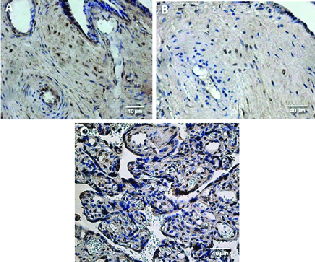
Apoptotic cells of the placenta. (A) Large tertiary villi of a 38 gestational-wk placenta with 1.64 ± 1.57 apoptotic cells of cytotrophoblast, syncytiotrophoblast, and extraembryonic mesoderm in a visual field (TUNEL, ×250). (B) Tertiary villi of a 28 gestational-wk placenta with 9.09 ± 4.93 apoptotic cells of extraembryonic mesoderm and cytotrophoblast in a visual field (TUNEL, ×250). (C) Tertiary villi of a transitional placenta of 40 gestational-wk with 43.91 ± 6.76 apoptotic cells of cytotrophoblast in a visual field (TUNEL, ×250).

Apoptotic cells were found in all placental samples of various gestational ages (Figure 2); in general, their amount decreased with advancing gestational age of the placenta. Apoptosis affected various cell types: cytotrophoblasts, syncytiotrophoblasts, extravillous trophoblasts, and cells of the extraembryonic mesoderm.

The mean number of apoptotic cells per visual field in the study groups were variable, with statistically significant (p < 0.01) differences between the term (13.00 ± 13.052) and preterm (27.00 ± 18.246) placentas. Besides, a statistically significant negative correlation was found between the number of apoptotic cells per visual field and the gestational age of the placenta (r = -0.278, p = 0.046). We also found that a higher number of pregnancies statistically significantly correlated with lesser number of apoptotic cells in term placentas (r = -0.529, p = 0.024). Also, a larger number of apoptotic cells in the placentas of the entire study significantly correlated with a smaller neonatal birth weight (r = -0.319; p = 0.021) and head (r = -0.277; p = 0.047) and chest (r = -0.310; p = 0.025) circumferences.

We also detected a positive correlation between the number of apoptotic cells and the graded value of HGF in the placentas of the entire study (r = 0.365, p < 0.01) and in the preterm placentas of the study (r = 0.420, p = 0.01).

**Table 1:** Maternal data


	**Minimum**	**Maximum**	**Mean ± Standard deviation**
**Maternal age (yr)**	18	39	29.79 ± 5.6
**Pregnancy (times)**	1	7	2.59 ± 1.64
**Delivery (times)**	1	6	1.75 ± 0.98

**Table 2:** Neonatal anthropometric parameters


	**Minimum**	**Maximum**	**Mean ± Standard deviation**
**Birth body weight (gr)**	540	4630	2367.15 ± 1122.59
**Body length (cm)**	28	59	45.51 ± 7.433
**Ponderal index (gr/cm ${}^{3}$)**	1.74	3.13	2.33 ± 0.31
**Head circumference (cm)**	22	39	31.15 ± 4.34
**Chest circumference (cm)**	20	37	29.19 ± 4.93

## 4. Discussion

As the placenta is a source of fetal nutrients and oxygen, placental status plays a key role in fetal well-being (19). Normal placental structure and morphometry (shape and size) indicate normal placental function, which provides growth of the fetus (20). Placental and fetal growth closely correlate with gestational age (21). Several studies have emphasized on a positive correlation between placental and birth weights (19, 22, 23). Similar to other studies, we found that the weight of both term and also pre-erm placentas correlated with birth weight. Low placental weight is associated with decreased placental surface area for gas and nutrient exchange, which may lead to fetal compromise (19). Souza and colleagues (24) investigated twin pregnancy and concluded that placental weight was lower in IUGR cases than in non-IUGR ones. Fetal growth restriction has been associated with adverse perinatal outcomes such as perinatal death, intrapartum distress, and low Apgar scores at birth (19). However, placental insufficiency is not only restricted to fetuses who are growth restricted, but also to those within the appropriate anthropometrical parameters (20).

We found that placental weight also correlated with the pre-pregnancy maternal weight moreover, with the weight gain during pregnancy. Lemas and colleagues (25) suggested that intrauterine exposure to maternal obesity influences fetal growth and fat increase. Higher placental weight is associated with gestational diabetes, fetal growth restriction, and maternal anemia (26). The association of an increased placental weight and adverse perinatal outcomes may indicate that the placenta tries to compensate with the surface area for a disturbed gas and nutrient transfer capacity (19).

There seemed to be less HGF-positive structures in placentas of more advanced gestational age, however, no statistically significant correlation was found. HGF occurrence negatively correlated with maternal weight gain in pregnancy. In literature, some studies have described an increasing expression of HGF in placentas with the advancement of the gestation in the 1${}^{\mathrm{st}}$ and 2${}^{\mathrm{nd}}$ trimesters of pregnancy (27), and also the HGF decrease in the amniotic membranes with advancing gestation and older maternal age (28). The rank value of HGF positively correlated with the number of apoptotic cells per visual field, suggesting regenerative efforts of the placental tissue. There are studies describing HGF to inhibit apoptosis in the placentas of pathological pregnancies, for example, in the case of preeclampsia (13, 29). Our observations also match with theses authors, suggesting that HGF promotes regeneration of tissues (30, 31). The absence of HGF in threatening situations could be interpreted as an indicator of placental decompensation.

Apoptosis is a process that is present in the placenta throughout pregnancy, like all the other live tissues, providing development (32). Apoptosis is regulated by the effector caspase pathway and the apoptosis inhibitor B-cell lymphoma 2 in the trophoblast (33). We found more apoptotic cells in preterm placentas in comparison with the term placentas. Our data is, however, inconsistent with the results of other studies, describing increased cellular apoptosis with advanced gestation (5). Their suggestions were based on a comparison of apoptotic cells in the third trimester with the first trimester, while we researched placentas only from the second and the third trimesters. Some other studies describe a significantly increased number of apoptotic cells in the placentas of pregnancies complicated by IUGR alone (34) or as a consequence of preeclampsia (35-38) or cigarette smoking (39). Superficial extravillous trophoblast invasion and poor spiral artery remodeling as a result of disturbances in the balance between extravillous trophoblast cell proliferation and cell death by apoptosis are seen in preeclampsia and IUGR (40, 41). Studies on IUGR pregnancies have pointed out that placental insufficiency may result in increased apoptosis and autophagy (33).

In this study, a significant negative correlation was found between the gestational age and the average number of apoptotic cells in a visual field, suggesting that in the third trimester of pregnancy, increased cellular apoptosis is seen in placentas with earlier termination. We suggest that cellular death in full-term placentas may change the balance between apoptotic and necrotic paths of cellular death. Necrosis, which is unprogrammed cell death, causes death in neighboring cells and induces inflammation. Abnormal fetal development may be induced by dysfunction of the placenta due to alterations in the differentiation, proliferation, and cell death (42).

At the end of the discussion, it has to be stressed that according to our observation, the weight of a preterm delivery placentas directly depended on the gestational age, maternal weight gain during pregnancy and correlated with the main fetal anthropometrical parameters (weight, length, head and chest circumferences) suggesting an impact of placental weight on the growth and development of fetus. The tendency of the decrease in number of HGF-positive cells seems to depend on the adaptation/ageing of placenta. We conclude that apoptosis is a feature of all placentas of various gestational ages and plays a crucial role in pregnancy continuation. Apoptotic cell number decreases with advanced gestation, suggesting a change in other ways of cellular disposal.

## 5. Conclusion

The weight of a placenta directly depends on the gestational age and correlates with the main fetal anthropometrical parameters (weight, length, head and chest circumferences).

The decrease in HGF-containing and apoptotic cells with advancing gestation depends on the adaptation potential of the placenta, proving the other ways of cellular disposition.

##  Conflict of Interest

None.
